# Significantly Elevated CA 19-9 after COVID-19 Vaccination and Literature Review of Non-Cancerous Cases with CA 19-9 > 1000 U/mL

**DOI:** 10.3390/jcm13051263

**Published:** 2024-02-23

**Authors:** Jakub Ciesielka, Krzysztof Jakimów, Natalia Tekiela, Laura Peisert, Anna Kwaśniewska, Dariusz Kata, Jerzy Chudek

**Affiliations:** 1 Student’s Research Group, Department of Internal Medicine and Oncological Chemotherapy, Medical University of Silesia in Katowice, 40-055 Katowice, Poland; krzysztof.jakimow@gmail.com (K.J.); s76501@365.sum.edu.pl (N.T.); s76008@365.sum.edu.pl (L.P.); 2 Department of Radiology, The Mielecki Hospital, Medical University of Silesia in Katowice, 40-055 Katowice, Poland; 3 Department of Hematology and Bone Marrow Transplantation, Medical University of Silesia in Katowice, 40-055 Katowice, Poland; dkata@wp.pl; 4 Department of Internal Medicine and Oncological Chemotherapy, Medical University of Silesia in Katowice, 40-055 Katowice, Poland

**Keywords:** antigen, CA 19-9, sialyl lewis A antigen, gastrointestinal cancer antigen, COVID-19 virus vaccines

## Abstract

Background: CA 19-9 is a commonly assessed tumor marker, considered characteristic of pancreatic ductal adenocarcinoma (PDAC) and biliary tract cancers; however, the positive predictive value of CA 19.9 is too low, and the usage of CA 19.9 as a screening tool in the healthy population remains controversial. Methods: The presented case illustrates a reversed diagnosis of highly elevated serum CA 19-9 levels in a 54-year-old female complaining of pain in the epigastric region, shortly after COVID-19 vaccination. Laboratory tests showed a significantly elevated level of the CA 19-9 marker (>12,000 U/mL, reference value: <37 U/mL) with normal pancreatic enzyme activity. The patient underwent imaging examination, which showed no abnormalities, except for increased pancreatic dimension and areas of fluid signal in the pancreas in magnetic resonance imaging (MRI), which may correspond to autoimmune pancreatitis (AIP). The patient remains asymptomatic with a recommendation for a follow-up MRI in 12 months. Results: A literature review conducted revealed multi-causal CA 19-9 increases above 1000 U/mL, including non-cancerous diseases of the lung, pancreas, liver, ovary, kidney, and others. The median concentration of CA 19-9 regardless of the cause of disease was 2810 U/mL (IQR ± 6895). The median CA 19-9 values in men and women were 3500 (IQR ± 10,050) and 2455 (IQR ± 3927), respectively, and differ significantly between the compared groups (*p* < 0.05). There was no difference between CA 19-9 values and the categorized cause of the increase. Conclusions: Conducting differential diagnosis, it should not be forgotten that most international guidelines recommend the use of CA 19-9 only in conjunction with pathology of pancreas in radiological imaging; however, even such a combination can point the diagnostic pathway in the wrong direction. A highly elevated CA 19-9 level, typically associated with PDAC, may be the result of benign disease including AIP related to COVID-19 vaccination.

## 1. Introduction

Physiologically, CA 19-9 (carbohydrate antigen 19-9, sialylated Lewis antigen A) is synthesized by normal epithelial cells of the gastrointestinal tract [1]. It is present in low amounts in serum and may be overexpressed in both benign and malignant gastrointestinal disorders [2]. High levels of CA 19-9 are considered to be characteristic of pancreatic and biliary tract malignancies; however, they have no diagnostic significance in patients without visualized pancreatic or biliary tract pathology [1]. The increased incidence of pancreatic ductal adenocarcinoma (PDAC) as well as the almost unchanged poor 5-year survival rate are the reasons for the increased CA 19-9 testing performed in laboratories by both patients and physicians [1,3]. We hereby present a case of reversed diagnosis of elevated serum CA 19-9 levels in a patient with chronic leucopenia vaccinated against COVID-19.

## 2. Materials and Methods

**Figure 1:**
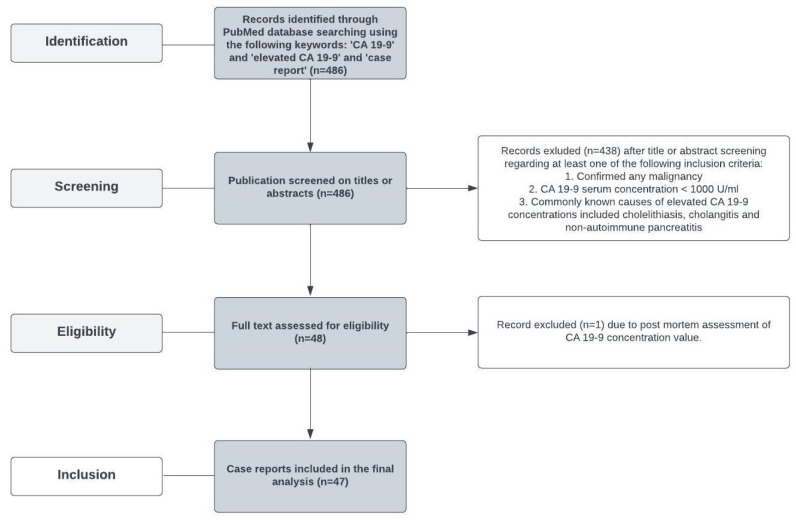
The flow chart for study selection.

To assess the non-cancerous causes of a significantly elevated CA 19-9 concentration, the PubMed database was searched using the following terms: ‘CA 19-9’ and ‘CA 19-9 elevation’ and ‘case report’. Then, 486 records were identified and screened on titles and abstracts. There were 439 patients who met at least one of the following criteria: any confirmed malignancy [1], CA 19-9 < 1000 U/mL [2]; common non-cancerous causes of CA 19-9 including cholelithiasis, cholangitis, and non-autoimmune pancreatitis [3] were excluded. Forty-eight case reports were assessed. A single case was excluded due to post-mortem CA 19-9 assessment. Finally, 47 cases were included in the final analysis. The flow chart for study selection is presented in Figure 1.

### 2.1. Data Extraction

The following data were extracted from each publication: sex, age, CA 19-9 serum concentration, confirmed disease, any observed decrease in CA 19-9 values after initial treatment (or follow-up), decrease or normalization of CA 19-9 values (reference range < 37 U/L), and time to normalization of CA 19-9 values. Identified diseases were categorized and assigned to 7 appropriate categories: lungs, bronchi, and mediastinum diseases [1]; spleen [2]; kidney [3]; ovary and uterus [4]; liver and biliary tract [5]; pancreas [6] and other diseases [7].

### 2.2. Statistical Analysis

All statistical analyses were performed using the Jamovi (Version 2.3, 2022) computer software. To assess the normal distribution, Shapiro–Wilk’s normality test was used. Continuous variables were expressed as mean (±SD) or median (±IQR) when appropriate. Categorical data were expressed as a number (percentage). Analysis of homogeneity of variance was performed using Levene’s test. The data with normal distribution were compared using a *t*-test. Analyzed data that were not normally distributed were compared using the U Mann–Whitney and ANOVA Kruskall–Wallis tests. Dwass–Steel–Critchlow–Fligner (DSCF) pairwise comparisons were used to compare age and CA 19-9 values assigned to a particular disease category. Statistical significance was determined as *p* < 0.05.

## 3. Case Report

A 54-year-old female with a medical history of chronic leukopenia was admitted to the hospital in October 2021 due to recurrent complaints of pain in the epigastric region that occurred in March 2021 as well as changes in the rhythm of bowel movements which were not accompanied by weight loss. In February 2021, she received a first dose of the AstraZeneca^®^ COVID-19 vaccine (chimpanzee adenovirus -ChAdOx1-S recombinant). Laboratory tests performed revealed elevated levels of CA125 (87 U/mL, reference value: <35 U/mL) and markedly increased CA 19-9 (>12,000 U/mL, reference value: <37 U/mL). A chest X-ray, gastroscopy, and colonoscopy revealed no pathology.

**Figure 2:**
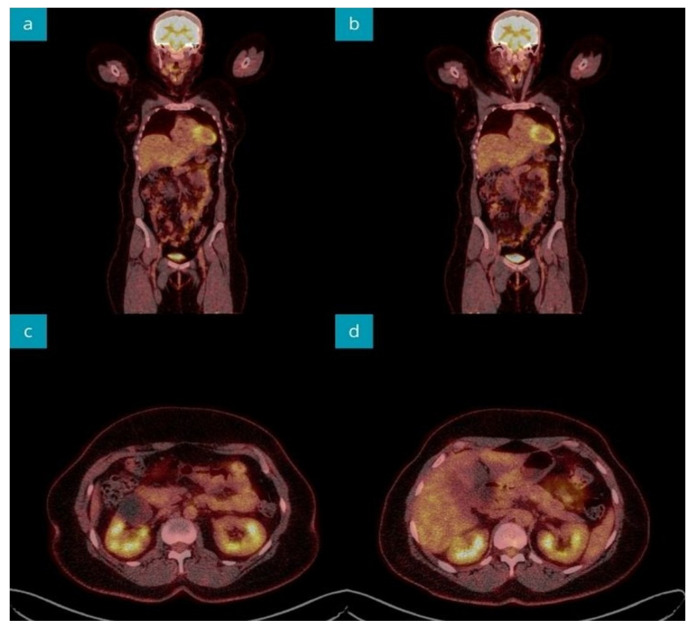
^18^F-FDG PET-CT imaging during the primary diagnostic process revealed abdominal organs without pathological ^18^F-FDG uptake. Coronal sections (**a**,**b**). Axial sections of pancreatic head (**c**), body, and tail (**d**).

A computed tomography (CT) scan of the abdomen disclosed a normal pancreatic image and hepatic steatosis; however, a blind liver biopsy showed no abnormalities. An abdominal nuclear magnetic resonance imaging (MRI) revealed a mildly increased pancreatic dimension with fine-point areas of fluid signal, accompanied by normal serum amylase and lipase activities. The patient underwent MR-cholangiopancreatography (MRCP) without visualizing biliary and pancreatic pathology. Due to the unclear image of the pancreas, the diagnostic work-up was completed by performing an ^18^F-fluorodeoxyglucose positron emission CT (^18^F-FDG PET-CT) imaging, which did not visualize pathological ^18^F-FDG uptake (Figure 2). The patient received the AstraZeneca^®^ COVID-19 booster in May 2019. At that time, no association between vaccination and pancreas pathology was established in the patient.

**Figure 3:**
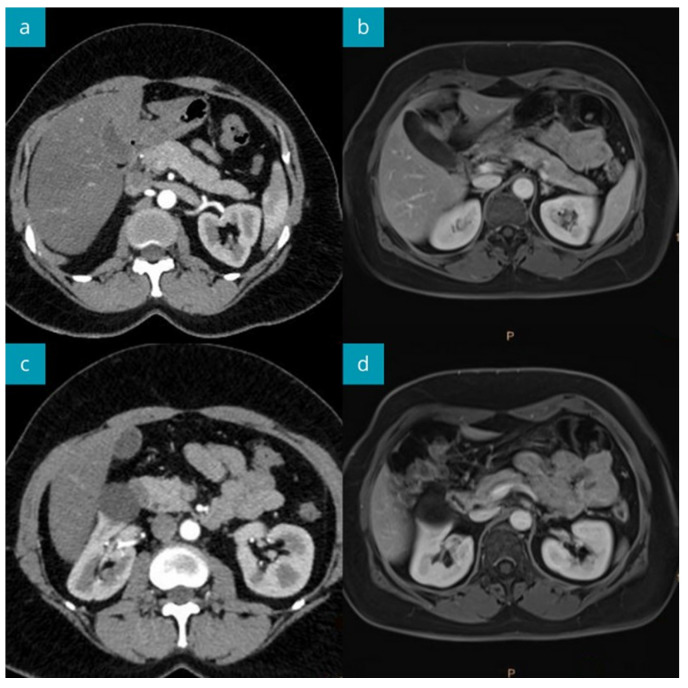
The abdominal CT imaging during the primary diagnostic work-up (**a**,**c**), and MRI after 15 months of follow-up (**b**,**d**). The head (mildly enlarged), body, and tail of the pancreas show normal structure. Normal pancreas image in MRI performed 15 months later.

Repeated MRI after 15 months revealed a normal pancreas image (Figure 3). The increased, but lower than previously, CA 19-9 value (1144 U/mL) was still observed in the asymptomatic patient.

## 4. Results

Forty-seven patients were finally included in the analysis, 17 men (36.2%) and 30 women (63.8%). Age values ranged from 13 to 80 years in the analyzed cohort with a median of 46 (IQR, 29–66.5). The median age of men and women was 52 (IQR, 38–70) and 42 (IQR 27–59.8), respectively (*p* = 0.24).

The median CA 19-9 concentration was 2810 U/mL (IQR, 1567–8461). The concentrations in men and women were 3500 (IQR, 1950–12,000) and 2455 (IQR, 1448–5365), respectively, and differed significantly between the compared groups (*p* < 0.05). The maximum noted CA 19-9 value was 96,554 U/mL. At diagnosis, 19.1% (n = 9) of patients were asymptomatic. Any decrease in CA 19-9 concentration after initial treatment was reported in 85.1% of all patients. The normalization of CA 19-9 values (stated as concentration < 37 U/mL) was observed in 63.8% of the cases. The median time to normalize CA 19-9 concentration values after the initial treatment was 2.0 months (IQR, 0.59–2.50). The detailed data are presented in Table 1.

The largest group consisted of patients with diseases of the ovary and uterus (n = 10; 21.3%), followed by: the pancreas (n = 8; 17.0%), liver, and biliary tract (n = 7; 14.9%), spleen (n = 7; 14.9%), lungs (n = 6; 12.8%), and kidneys (n = 4; 8.5%). Five patients (10.6%) were not assigned to any category. Analyzing anthropometric and CA 19-9-related data depending on the disease category, the highest median age was observed in patients with liver and biliary tract diseases (70, IQR 58–71). The highest percentage of asymptomatic patients was 60% in the group with other diseases which included: rheumatoid arthritis (RA), *Helicobacter pylori* infection, giardiasis, and diaphragmatic cyst, followed by patients with ovary and uterus pathology, where this percentage was 40%. The highest CA 19-9 values were noted in patients with liver and biliary tract pathology, where the median value was 6000 U/mL (IQR, 2315–15,316). Any decrease in CA 19-9 values was noted in all patients with ovary and uterus, kidney, liver, and biliary tract diseases. Normalization of CA 19-9 concentration was noted in all patients with kidney diseases. The age of patients assigned to the particular disease category varied significantly (*p* = 0.001) between the compared disease categories. DSCF pairwise comparisons revealed that the age of patients with ovarian diseases differed significantly compared to the age of patients with liver (*p* < 0.05) diseases. The CA 19-9 values did not differ by disease category (*p* = 0.36). Detailed data are presented in Table 2.

## 5. Discussion

CA 19-9 is a human glycoprotein widely synthesized in the pancreatic, biliary tract, gastric, colon, endometrial, and salivary gland cells [1]. Elevated CA 19.9 levels are commonly associated with pancreatic and biliary tract cancer, in which the prognosis and 5-year survival remain poor [1,48]. Serum CA 19-9 concentration is raised in more than 80% of patients with advanced pancreatic cancer and is routinely used to monitor the course of the disease, both during treatment and follow-up. Attempts to use CA 19-9 as a screening tool for pancreatic cancer in the general population have failed because of the low positive predictive value of that test, which does not exceed 90% [49]. However, higher CA 19-9 values should raise a suspicion of malignancy [50]. Notwithstanding, approximately 6% of Caucasian patients and 22% of non-Caucasian patients do not synthesize the Lewis antigen, the presence of which determines the CA 19-9 antigen, so there is a significant risk of false-negative results in this subgroup of patients, even with highly advanced-stage pancreatic cancer [1,51].

CA 19-9 was proved to have limited screening utility even in high-risk populations, such as patients with familial pancreatic ductal adenocarcinoma or Peutz–Jeghers syndrome. In these populations, CA 19-9 levels were normal even when imaging revealed preinvasive lesions [52]. However, it should be stressed that for diagnostic purposes, most international guidelines recommend the use of CA 19-9 in conjunction with imaging examinations such as pancreatic CT, which is now the gold standard for diagnosing pancreatic lesions [53]. 

The median sensitivity and specificity of CA 19-9 in the prediction of pancreatic adenocarcinoma with a generally accepted cut-off point of 37 U/mL are 79% and 82%, respectively, with a positive predictive value of 72% and negative predictive value of 81%, indicating a relatively large percentage of potentially false-positive results [54]. It has been proven that values of CA 19-9 below 100 U/mL characterize potentially resectable tumors, whereas higher values (above 100 U/mL) are more typical for unresectable and aggressive tumors, higher rates of recurrence, and poor outcomes [55]. The specificity of prediction of PDAC in patients with values above 100 U/mL is 98%, whereas CA 19-9 levels of more than 1000 U/mL are strongly associated with unresectable pancreatic cancer with the specificity of detection reaching 100% [56,57]; however, cases of patients with CA 19-9 exceeding 1000 U/mL and co-occurring benign diseases have also been reported in the literature (Table 1). 

In the performed statistical analysis, no relevant differences were noted between CA 19-9 levels and gender as well as disease category. The age values of patients presenting CA 19-9 elevations above 1000 U/mL with diagnosed ovary and uterine diseases differed significantly compared to patients with liver diseases. This can be explained by a high proportion of ovarian pathologies in relation to other diseases and the dominance of teratomas, endometriomas, and epidermoid cysts in it, which usually occur in young and middle-aged women when liver pathologies including Mirizzi syndrome, inflammatory pseudotumor, and liver abscesses are most common in the population over 60 years of age. Most of the analyzed patients presented in Table 1 were symptomatic at the time of CA 19-9 evaluation; however, we found nine cases of asymptomatic patients who presented with elevated CA 19-9 above 1000 U/mL. de Meira Junior et al. [47] reported a case of an asymptomatic 52-year-old male with elevated CA 19-9 to 96,544 U/mL, revealed during routine testing. Performed CT, MRCP, and PET-CT scans showed no abnormalities. One month after primary testing, the CA 19-9 value decreased to 2822 U/mL, and after 4 months from the primary testing, CA 19-9 values were within the reference range (<37 U/mL) [47]. Sheng-Che L. et al. [23] reported a case of a 27-year-old woman who was referred to whole-body PET-CT due to persistently elevated CA 19-9 to values of 3498 U/mL. Pathologically ovarian dermoid cyst was diagnosed [23]. Cho A. et al. [24] reported a case of a 26-year-old woman with an ovarian cyst discovered during a routine gynecological examination with CA 19-9 levels of 1634 U/mL. Two months after surgery, CA 19-9 levels decreased to 64 U/mL [34]. 

Recent reports have indicated that COVID-19 vaccinations using mRNA or viral vector vaccines can trigger autoimmune diseases such as sialadenitis, autoimmune hepatitis (AIH), or autoimmune pancreatitis (AIP) in selected individuals [58,59,60,61,62,63,64,65]. In the literature, several reports have treated the correlation between autoimmune pancreatitis and COVID-19 vaccination (Table 3).

The occurrence of AIP has been mostly found in males, interestingly, after the second dose of the vaccine. The primary dose might not have been the adequate trigger factor to cause the autoimmune process; thus, the AIP occurred after the booster vaccination. Patients with the new onset of AIP were mostly not diagnosed previously with any autoimmune diseases. An elevation of CA 19-9 values was found in none of the patients. 

Several conceptions have been postulated in the literature to explain the connection between COVID-19-mRNA vaccines and the occurrence of AIP [66,67]. According to the first hypothesis, the are many molecular similarities between the products of mRNA viral vaccine and viral proteome; thus, the mechanism leading to AIP could be the same as in viral infection. In the literature, authors postulate that AIP during COVID-19 infection may be caused by a too high affinity of viral glycoproteins to the angiotensin II receptor, highly expressed in the pancreatic tissue, consequently triggering an autoimmune process [59,66]. According to other hypotheses, the molecular mimicry between the viral glycoproteins and autoantigens could be a trigger for the synthesis of cross-reacting antibodies, leading to autoimmune diseases [59,67]. 

The patient described in this case report suffered from recurrent epigastric pain and a change in bowel movements. Her CA 19-9 level was markedly elevated, reaching values of over 12,000 U/mL. Herein, we report six cases of patients with AIP, causing the elevation of CA 19-9 ranging from 1950 U/mL to 12,000 U/mL, and a case of a patient with liver steatosis and an increased CA 19-9 value to 1930 U/mL. When considering differential diagnosis due to the low specificity of tumor markers and lack of obvious radiological image, various diseases should be taken into consideration [4,5,6,7,8,9,10,11,12,13,14,15,16,17,18,19,20,21,22,23,24,25,26,27,28,29,30,31,32,33,34,35,36,37,38,39,40,41,42,43,44,45,46,47,68,69]; however, a mildly increased dimension of the pancreas in MRI with areas of fluid signals could correspond to past AIP that induced increased CA 19-9. According to the literature, about 25% of patients with AIP show values of CA 19-9 > 37 U/mL, and only around 12.2% of them show elevated levels > 100 U/mL [70]. Considering the time coincidence, we hypothesize that vaccination with the use of a COVID-19 vector recombinant vaccine might have been an inciting event in our patient, triggering possible autoimmune processes, finally leading to increased production and secretion of CA 19-9. Moreover, there are reports on leukopenia and the induction of AIP. We postulate that chronic leukopenia may have contributed to an impaired immune response and interacted synergistically with vaccination in the induction of AIP. Due to the self-limiting inflammatory process and rapid resolution of symptoms after the diagnostic process, steroid therapy was abandoned. 

Five main features of AIP should be taken into consideration: radiological imaging, histology of the pancreas, serum IgG4, other organ involvement, and response to steroid therapy [70,71,72]. Radiological imaging is essential for stating the diagnosis of AIP. In CT, the pancreas is described as diffusely swollen with a diffusely or segmentally narrowed pancreatic duct (Criterion I). Criterion II and Criterion III consist of elevated levels of IgG4 in the serum or detected autoantibodies and histopathologic findings such as fibrosis and lymphoplasmocytic infiltration, respectively. A correlation with other autoimmune diseases is considered as Criterion IV. The diagnosis is definite when Criteria I and II/III are met or if Criteria I and IV are met when an answer to steroid therapy is present. Meeting only Criterion I suggests AIP as a possible solution [72]. Table 4 presents revised criteria for AIP.

Progressive AIP may result in steatorrhea or diabetes mellitus (DM) because of both exocrine and endocrine pancreatic dysfunction [73]. DM occurs in 52% of newly diagnosed patients with AIP; however, according to some studies, DM may affect up to 78% of patients with AIP [74,75]. Response to steroid therapy may be achieved in 55% of patients with AIP and newly diagnosed DM [75]. Patients may also develop pancreatic duct stones, cirrhosis, and portal hypertension. There is also an increase in malignancy occurrence regarding stomach, lung, and prostate cancers [73]. 

MRI revealed a mildly enlarged pancreas, which is an inclusion and essential criterion for AIP [73], with fine-point fluid collections. That sign has to be differentiated between other forms of pancreatic diseases including acute pancreatitis, PDAC, or AIP. When analyzing an MRI of the mildly increased pancreas, radiologists should pay attention to several subtle phenomena that occur in AIP that are considered high-accuracy signs in the differentiation between AIP and PDAC. These images include enhanced duct and capsule-like rim signs as well as punctuating enhancements within the lesion [76]. 

In our study, an abdominal MRI disclosed a normal pancreatic duct; however, according to some authors, the nondilated pancreatic duct can be observed in some patients with AIP [70]. In addition to MRCP, serum IgG4 level, and histology of the pancreas being emphasized in the AIP diagnostic guidelines [70,71,72,73], the additional examination was not performed due to the good clinical condition of the patient. Scheduled abdominal MRI and biochemical work-up will allow for control of the radiological image of the pancreas as well as assess the dynamics of CA 19-9 concentration changes and, if necessary, implement appropriate management.

## 6. Conclusions

We postulate that reversed diagnosis can be of great value, leading to a possible answer to a problem, but it can also draw focus to an improper diagnosis. CA 19-9 concentrations > 1000 U/mL can occur in numerous non-cancerous diseases. In the presented case, an elevated concentration of CA 19-9 > 12,000 U/mL, typically associated with gastrointestinal tract malignancies, was the result of a benign disease—probably an autoimmune pancreatitis triggered by a COVID-19 vaccination. We suggest that future studies assessing mRNA vaccine safety should include a rare adverse event—AIP.

## 7. Limitations

A potential limitation may be inherent to our paper. In the course of the diagnostic work-up, the priority was to exclude malignancy. In the clinical course of a self-limiting inflammatory process, resulting in the resolution of symptoms, the assessment of IgG4 antibodies was abandoned. Nevertheless, pancreatic imaging, temporal coincidence with vaccination, and chronic leukopenia make AIP the most probable diagnosis. 

## Figures and Tables

**Table 1:** Characteristics of patients with elevated CA 19-9 serum concentration > 1000 U/mL, meeting the criteria for inclusion in the analysis.

Sex	Age	Symptoms Prior to Diagnosis (Yes, No)	CA 19-9 Serum Concentration [U/mL]	Final Diagnosis	Any CA 19-9 Decrease after Treatment (Yes, No)	CA 19-9 Normalization after Treatment (<37 U/mL) (Yes, No)	Time to Normalization of CA 19-9 after Therapy (Days)	Reference
Lungs, bronchi, and mediastinum diseases								
Female	80	Yes	1118	Interstitial pulmonary disease	NA	NA	NA	[4]
Male	57	Yes	1300	Bronchogenic cyst	Yes	Yes	0.5 month	[5]
Female	48	Yes	>1200	Bronchogenic cyst	Yes	Yes	2 months	[6]
Male	39	Yes	3051.1	Pulmonary sequestration	Yes	Yes	6 months	[7]
Female	46	Yes	2810.3	Pulmonary sequestration	Yes	Yes	3 months	[8]
Female	67	Yes	>1500	Bronchiectasis	Yes	No	No	[9]
Spleen diseases								
Female	20	Yes	43,000	Epidermoid splenic cyst	Yes	NA	NA	[10]
Female	23	Yes	17,580	Epidermoid splenic cyst	Yes	Yes	4 months	[11]
Female	33	Yes	3347	Epidermoid splenic cyst	Yes	Yes	0.5 month	[12]
Male	25	Yes	2878	Epidermoid splenic cyst	Yes	Yes	0.68 month	[13]
Male	52	Yes	1918	Epidermoid splenic cyst	Yes	Yes	2 months	[14]
Female	24	Yes	1200	Epidermoid splenic cyst	NA	NA	NA	[15]
Male	16	Yes	1264	Epidermoid splenic cyst	Yes	Yes	0.23 month	[16]
Kidney diseases								
Male	58	Yes	3500	Hydronephrosis	Yes	Yes	2 months	[17]
Female	56	No	3049	Hydronephrosis	Yes	Yes	6 months	[18]
Female	42	Yes	2500	Hydronephrosis	Yes	Yes	NA	[19]
Female	74	Yes	>1000	Hydronephrosis	Yes	Yes	NA	[20]
Ovary and uterine diseases								
Female	25	No	1430	Epidermoid cyst	Yes	Yes	NA	[21]
Female	50	Yes	8922.76	Teratoma	Yes	Yes	NA	[22]
Female	27	No	3498	Teratoma	Yes	Yes	6 months	[23]
Female	26	No	1633.68	Teratoma	Yes	No	No	[24]
Female	37	Yes	2753	Mucinous cystadenoma	Yes	Yes	NA	[25]
Female	55	Yes	1999	Mucinous cystadenoma	Yes	Yes	2 months	[26]
Female	27	Yes	7946	Ovarian endometrioma	Yes	Yes	NA	[27]
Female	34	Yes	7604	Ovarian endometrioma	Yes	Yes	1.5 months	[28]
Female	39	No	1796	Denomyoma	Yes	Yes	NA	[29]
Female	13	Yes	>1000	Hydrometrocolpos	Yes	Yes	0.5 month	[30]
Liver and biliary tract diseases								
Male	71	Yes	21,068	Mirizzi syndrome	Yes	No	NA	[31]
Male	71	Yes	>16,000	Mirizzi syndrome	Yes	Yes	0.16 month	[32]
Female	72	Yes	14,632	Liver inflammatory pseudotumor	Yes	Yes	1 month	[33]
Male	50	Yes	1167.9	Liver inflammatory pseudotumor	Yes	Yes	1 month	[34]
Female	66	Yes	6000	Pyogenic liver abscess	Yes	Yes	2 months	[35]
Female	45	Yes	1930	Hepatic steatosis	Yes	No	NA	[36]
Female	70	Yes	2700	Biliary cystadenoma	Yes	Yes	2 months	[37]
Pancreas diseases								
Male	38	Yes	>12,000	Autoimmune pancreatitis	Yes	No	NA	[38]
Male	72	Yes	>12,000	Autoimmune pancreatitis	Yes	No	1 month	[38]
Male	72	Yes	>10,000	Autoimmune pancreatitis	Yes	NA	NA	[39]
Male	70	Yes	8000	Autoimmune pancreatitis	NA	NA	NA	[38]
Male	31	Yes	3282	Autoimmune pancreatitis	NA	NA	NA	[40]
Male	69	Yes	1950	Autoimmune pancreatitis	NA	NA	NA	[38]
Male	25	Yes	18,860	Pancreatic tuberculosis	NA	NA	NA	[41]
Female	26	No	1036	Heterotopic pancreas	Yes	NA	NA	[42]
Others								
Female	61	Yes	12,000	Diaphragmatic epidermoid cyst	Yes	Yes	3 months	[43]
Female	42	No	2410	Giardiasis	Yes	Yes	0.32 month	[44]
Female	36	No	1880	H. Pylori infection	Yes	Yes	NA	[45]
Female	70	Yes	1000	Rheumatoid polyarthritis with pulmonary fibrosis	NA	NA	NA	[46]
Male	52	No	96,544.3	No etiological factor was found	Yes	No	NA	[47]

NA—not available.

**Table 2:** Anthropometric and CA 19-9-related data stratified on the disease category.

Disease Category	Female; *n* (%)	Male; *n* (%)	Median Age	Median CA 19-9 Values
Lungs, bronchi, and mediastinum	4 (66.7)	2 (33.3)	52.5 (IQR, 46.5–52.5)	1400 (IQR, 1225–2483)
Spleen	4 (57.1)	3 (42.9)	24 (IQR, 21.5–29)	2878 (IQR, 1591–10,464)
Kidney	3 (75)	1 (25)	57 (IQR, 52.5–62)	2775 (IQR, 2125–3162)
Ovary and uterus	10 (100)	0 (0)	30.5 (IQR, 26.3–38.5)	2376 (IQR, 1674–6578)
Liver and biliary tract	4 (57.1)	3 (42.9)	70 (IQR 58–71)	6000 (IQR, 2315–15,316)
Pancreas	1 (12.5)	7 (87.5)	53.5 (IQR, 29.8–70.5)	9000 (IQR, 2949–12,000)
Others	4 (80)	1 (20)	52 (IQR, 42–61)	2410 (IQR, 1880–12,000)

**Table 3:** The literature reports on AIP triggered by COVID-19-mRNA vaccines.

Sex	Age	Vaccine	The Onset of Symptoms after the Primary (P) or Secondary (S) Dose; Time Interval from Vaccination	Confirmed Other Autoimmune Diseases	Confirmed Elevation of CA 19-9	Ref.
M	63	COVID-19-mRNA	S; 60 days	no	no	[58]
M	39	Gam-COVID-Vac (Sputnik V)	S; 4 days	no	no	[59]
M	54	Pfizer/BioNTech COVID-19 mRNA	S; 30 days	yes	no	[60]
M	65	Pfizer/BioNTech COVID-19 mRNA	P; 14 days	no	no	[61]
F	78	Pfizer/BioNTech COVID-19 mRNA	S; 14 days	no	no	[62]

**Table 4:** The revised criteria for AIP diagnosis and case presentation. Adapted from Kim KP et al. Diagnostic criteria for autoimmune chronic pancreatitis revisited. World Journal of Gastroenterology **2006**, 12, [72].

Autoimmune Chronic Pancreatitis (AIP) Diagnostic Criteria		Presented Case
Criterion I: Pancreatic Imagining	CT: Diffuse enlargement (swelling) of the pancreas	Yes
	ERCP: Diffuse or segmental irregular narrowing of the main pancreatic duct	No
Criterion II: Laboratory Findings	Elevated levels of IgG and/or IgG4	No data
	Detected autoantibodies	No data
Criterion III: Histopathologic findings	Fibrosis and lymphoplasmacytic infiltration	No data
Criterion IV: Association of other postulated autoimmune disease		No
Definite: I + II + III + I or I + II + III or I + II/I + III Probable: I + IV (Rediagnosed as ‘’definite” if respond to steroid is present) Possible: Only I		

## Data Availability

Additional patient data can be obtained from the authors upon reasonable request.
